# Looking Beyond the Surface: Olutasidenib and Ivosidenib for Treatment of m*IDH1* Acute Myeloid Leukemia

**DOI:** 10.1007/s11864-024-01264-7

**Published:** 2024-10-16

**Authors:** Justin M. Watts, Simon J. Shaw, Brian A. Jonas

**Affiliations:** 1 Sylvester Comprehensive Cancer Center, Division of Hematology, University of Miami, 1475 Northwest 12th Ave, Miami, FL 33136 USA; 2 Rigel Pharmaceuticals, Inc., South San Francisco, CA USA; 3 UC Davis Comprehensive Cancer Center, University of California, Davis Sacramento, CA USA

**Keywords:** Isocitrate dehydrogenase, Mutant IDH1, Alpha-ketoglutarate, 2-hydroxyglutarate

## Abstract

Mutations in isocitrate dehydrogenase-1 (*IDH1)* are recurrent in several malignancies and prevalent in acute myeloid leukemia (AML). Olutasidenib and ivosidenib are inhibitors that target mutant IDH1 (mIDH1) and are FDA approved for the treatment of patients with m*IDH1* AML*.* Olutasidenib and ivosidenib were identified through unique molecular screens and thus are structurally very different molecules. A difference in clinical outcomes has been observed with olutasidenib, which has a longer duration of response than ivosidenib, despite similar rates of response being achieved with the two drugs, such as complete remission (CR) or CR with partial hematologic recovery (CR/CRh). In the absence of a head-to-head trial, this review examines both the extent of differences in clinical outcomes with the two drugs and provides the first comparison of the unique molecular and mechanistic features of each drug, such as molecular structure and binding kinetics, that may contribute to the observed clinical difference in outcomes. Olutasidenib is structurally smaller with a lower molecular weight than ivosidenib (FW 355 vs FW 583) and thus occupies less space in the binding pocket of IDH1 dimers, making it resistant to displacement by IDH1 second-site mutations. In biochemical studies, olutasidenib selectively inhibits mutant but not wild-type IDH1, whereas ivosidenib appears to potently block both mutant and wild-type IDH1. Although they have the same target, olutasidenib and ivosidenib have unique molecular features, which may translate to selectivity differences in their inhibitory activity against IDH1.

## Introduction

Acute myeloid leukemia (AML) is characterized by loss of differentiation and uncontrolled proliferation of immature myeloblasts. Treatment of AML has undergone recent advancements based on molecular analysis identifying recurrent mutations that are targets for therapy [1]. One such genomic target leading to recent FDA approvals in AML is mutated *IDH1* R132 (isocitrate dehydrogenase 1), which plays a role in tumorigenesis through its involvement in the dysregulation of DNA methylation, metabolism, and other cellular functions [2, 3]. Mutations in *IDH1* are recurrent in several malignancies and prevalent in AML.

Wild-type (wt) IDH1 function is critical for normal cellular metabolism, catalyzing conversion of isocitrate to α-ketoglutarate (α-KG) and CO_2._ Mutant forms of IDH1 (mIDH1) have altered or gain-of-function enzymatic activity, leading to a neomorphic reaction that causes reduction of α-KG to the oncometabolite, 2-hydroxyglutarate (2-HG), and depletion of NADPH [2, 3]. The abnormal production of 2-HG blocks cellular differentiation and promotes tumorigenesis. The mutant *IDH1* R132, by virtue of poor binding to isocitrate, has reduced capability to generate α-KG [2].

Approximately 7–14% of AML patients harbor an *IDH1* mutation [4]. Since the characterization of mIDH1 in AML, selective oral inhibitors that target mIDH1 have been developed, and olutasidenib and ivosidenib are now FDA approved for the treatment of patients with m*IDH1* AML [5, 6]. Although both drugs bind IDH1, the two molecules were developed through different drug screening processes and their molecular structures are different having originated from distinct chemical scaffolds.

Olutasidenib and ivosidenib have both demonstrated clinical efficacy in patients with m*IDH1* AML [7, 8]. Differences in efficacy have been observed in their respective clinical studies; although notably the two drugs have not been compared in a head-to-head trial. The aim of this review is to examine not only the extent of differences in clinical response between the two drugs but also unique molecular and mechanistic features, such as molecular structure and binding kinetics, that may contribute to their individual clinical profiles.

## Clinical Summary of Olutasidenib and Ivosidenib

Olutasidenib monotherapy administered at 150 mg orally, twice daily, was evaluated in a pivotal phase 2 cohort of 153 patients with relapsed/refractory (R/R) AML harboring mIDH1 (NCT02719574; study started April 2016) [7]. In this trial, patients had a median age of 71 years and a median of 2 prior regimens before starting the trial. Over a median follow-up of 10.2 months, the complete remission (CR) plus CR with hematologic recovery (CR/CRh) rate was 35%, and the median duration of CR/CRh response was 25.9 months [7]. The overall response rate (ORR) was 48% for a median duration of overall response of 11.7 months [7]. The median overall survival (OS) was 11.6 months. There were 16 R/R AML patients who proceeded to HSCT after responding to olutasidenib monotherapy [9].

Ivosidenib monotherapy given orally at 500 mg daily was evaluated in a phase 1 dose escalation cohort of 179 patients with R/R AML with an *IDH1* mutation (NCT02074839; study started March 2014) [8]. In this trial, patients had a median age of 67 and a median of 2 prior therapies. The CR/CRh rate in the primary efficacy population (*n* = 125) was 30%, with a median duration of CR/CRh of 8.2 months [8]. The ORR was 42% for R/R AML and median duration of ORR was 6.5 months [8]. The median OS was 8.8 months during a median follow-up period of 14.8 months [8]. There were 17 patients who responded to ivosidenib treatment and proceeded to HSCT [10].

With the caveat that comparing results from two independent studies conducted in sequence at slightly different times should be done with caution, there was a notably longer duration of response observed in the olutasidenib trial, with a difference in median duration of CR/CRh of almost 18 months (Table 1). The difference in DOR among CR/CRh responders cannot be explained by the number of patients who were able to proceed to HSCT and thus discontinued the study since both studies continued to be followed for DOR, as neither study was censored for HSCT. Furthermore, although the rate of CR/CRh was similar between the two studies, there was a higher proportion of patients who achieved a complete remission with olutasidenib (32% vs 22%). Possible reasons for the differences observed include study design (discussed above), patient characteristics, and molecular characteristics. The baseline characteristics of the two studies are summarized in Table 2.

**Table 1:** Clinical efficacy in R/R AML patients receiving monotherapy in the primary efficacy populations

	Olutasidenib *N* = 147^a^	Ivosidenib *N* = 125^b^
CR/CRh	35%	30%
CR	32%	22%
CRh	3%	8%
Duration of CR/CRh in months, median (95% CI)	25.9 (13.5, NR)	8.2 (5.5, 12)

^a^The olutasidenib primary efficacy population consisted of patients with R/R AML who had centrally confirmed mIDH1. The median follow-up was 10.2 months

^b^The ivosidenib primary efficacy population consisted of patients with R/R AML who received 500 mg ivosidenib daily and had at least 6 months of follow-up. The median follow-up was 14.8 months

**Table 2:** Demographics and characteristics [7, 8]

	Olutasidenib *N* = 147^a^	Ivosidenib *N* = 125^a^
Age, median (range)	71 (32–87)	67 (18–87)
Male, *n* (%)	74 (50)	65 (52)
AML type, *n* (%)		
De novo	97 (66)	83 (66)
Secondary	50 (34)	42 (34)
Cytogenetic risk, *n* (%)		
Favorable	6 (4)	0
Intermediate	107 (73)	66 (53)
Poor	25 (17)	38 (30)
Missing/unknown	9 (6)	21 (17)
Co-mutations, *n* (%)^b^		
NPM1	31 (21)	24 (20)
FLT3	15 (10)	9 (8)
TP53	9 (6)	NA
Prior AML therapy outcome, *n* (%)		
Primary refractory	46 (31)	64 (37)^c^
Untreated relapse	81 (55)	65 (37)^c^
Refractory relapse	20 (14)	45 (26)^c^
Prior Regimens, median (range)	2 (1–7)	2 (1–6)
Venetoclax, *n* (%)	12 (8)	-
HSCT, *n* (%)	17 (12)	36 (29)
ECOG PS, *n* (%)		
0	45 (31)	27 (22)
1	76 (52)	64 (51)
2	23 (16)	32 (26)
3	0	2 (2)

^a^Primary efficacy population

^b^*n* = 119 for IVO

^c^*n* = 174 for full IVO cohort (from Tibsovo® prescribing information)

When examining the baseline patient characteristics from these two studies, the ivosidenib population had a higher percentage of patients with poor-risk cytogenetics (30% vs 17% for olutasidenib) and refractory relapse (26% vs. 14% for olutasidenib), as well as a higher number of patients who received prior hematopoietic stem cell transplantation (29% vs 12% for olutasidenib). On the other hand, the olutasidenib cohort was a more heavily pretreated patient population, with 68% of patients in the olutasidenib study having 2 or more prior lines of systemic therapies compared to 59% in the ivosidenib cohort. Additionally, due to the time periods of the two studies, only the olutasidenib population included patients that were exposed to prior venetoclax (12 patients from the pivotal cohort) [11], which is a particularly poor prognosis group [12]. Venetoclax was not available at the time of the ivosidenib study. The ivosidenib patient population also had a younger median age than the olutasidenib patient population, with the former enrolling patients as young as 18 years old and the latter enrolling ≥ 32 years old. In the olutasidenib population, there were 45 patients aged 75 years and older.

These modest differences seen in baseline characteristics may have influenced response rates [13, 14], but there are no reports of patient or baseline characteristics predicting duration of response. When examining available data, patients with similar baseline characteristics (e.g. mutation status, disease features, and patient-related factors) appear to have similar response rates to either IDH1-targeted therapy [7, 8]; however, there is a less clear association between any particular patient or disease characteristic and the duration of response to either therapy. Patient-specific characteristics are thus unlikely to explain the marked difference in duration of response between the two drugs. It is therefore of compelling interest to further explore and compare the distinct molecular, chemical, and pharmacokinetic/pharmacodynamic features of these agents to determine the rationale behind the longer duration of response observed with olutasidenib. Moreover, not only does duration of response correlate with overall survival, but it is directly dependent on the primary therapy under investigation, not reflecting any possible impact on OS by subsequent therapy given after IDH1 inhibitor treatment failure.

## Biological and Chemical Properties of Olutasidenib and Ivosidenib

**Fig. 1:**
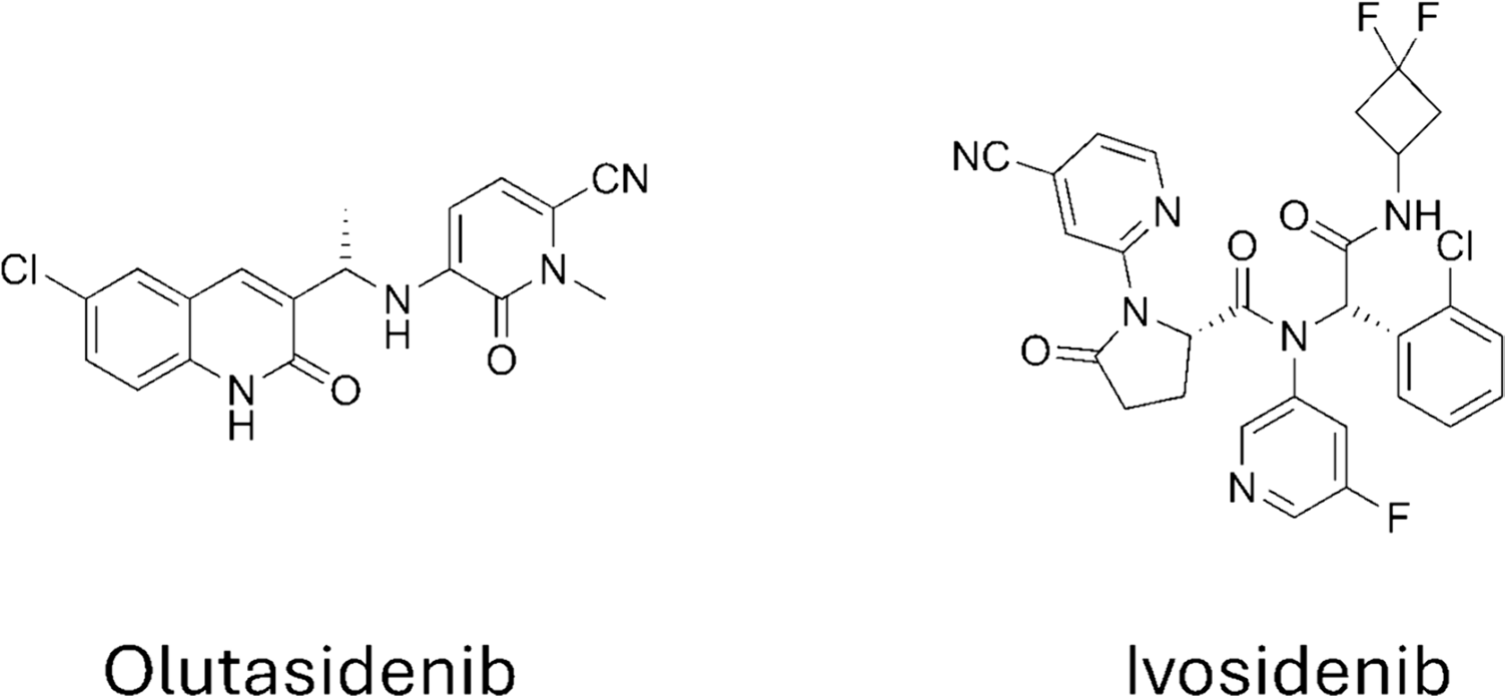
Molecular structure of olutasidenib and ivosidenib. (left) Olutasidenib has a chemical formula of C_18_H_15_CIN_4_O_2_. The molecular weight of olutasidenib is 355 g/mol. (right) Ivosidenib has a chemical formula of C_28_H_22_CIF_3_N_5_O_3_. The molecular weight of ivosidenib is 583 g/mol

Although ivosidenib and olutasidenib inhibit the same target, namely the mutant IDH1 protein dimer, they are structurally distinct molecules with different aspects to their respective specificity and binding mechanisms (Fig. 1).

### Selectivity for Mutant vs. Wild-Type IDH1

In comparing the individual pharmacokinetic/pharmacodynamic profiles of the two molecules, one key difference between olutasidenib and ivosidenib involves their selectivity. While both molecules are effective at inhibiting mutant IDH1, olutasidenib exhibits greater selectivity towards targeting the mutant form without inhibiting wild-type IDH1. In vitro studies showed that olutasidenib inhibited mutated IDH1 R132H, R132L, R132G, and R132C proteins; however, wild-type IDH1 and mutated IDH2 proteins were not inhibited. The inhibition of mutant IDH1 by olutasidenib led to reduced 2-HG levels. In contrast, ivosidenib potently inhibits both mutant and wild-type isoforms of IDH1. The IC50 of olutasidenib for wild-type IDH1 is 22,400 nM in stark contrast to the IC50 of ivosidenib for wild-type IDH1, which is 24-71 nM [15, 16]. Thus, ivosidenib inhibits wild-type IDH1 at an almost 1000-fold lower concentration [15].

**Fig. 2:**
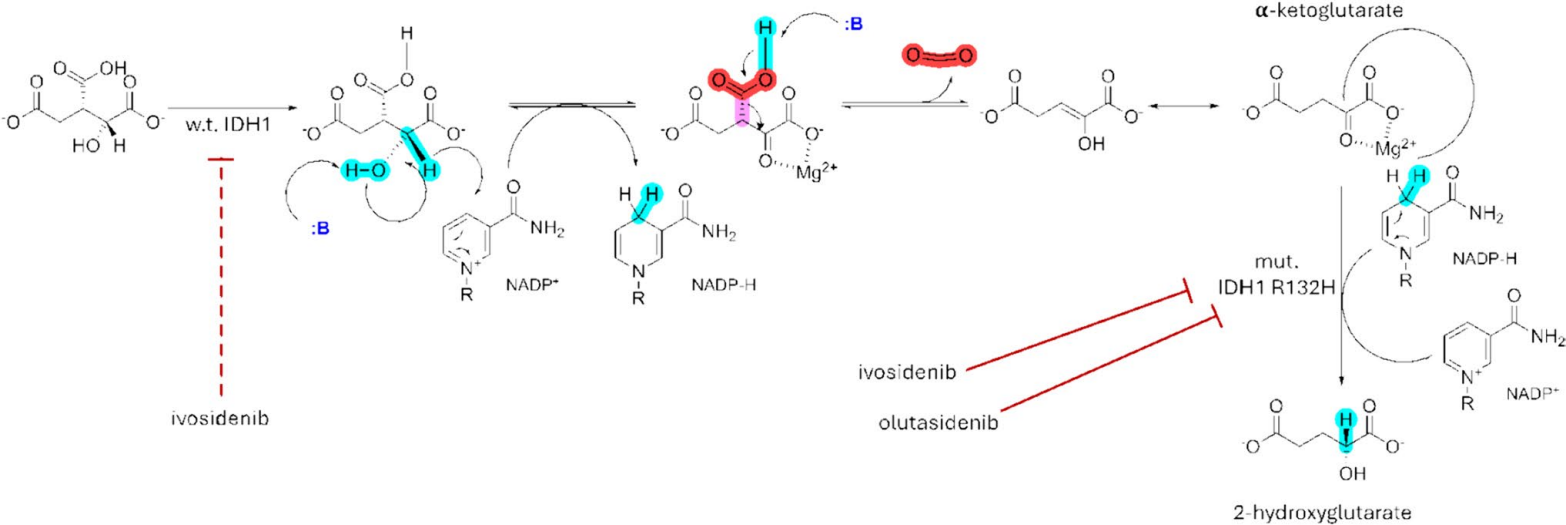
Reactions catalyzed by wild-type and mutant IDH1. Wild-type IDH1 (w.t. IDH1) converts isocitrate to α-ketoglutarate (α-KG) in a reaction that uses NADP + as an electron acceptor and produces NADPH. A point mutation in the *IDH1* gene at the Arg132 residue (R132) results in a gain-of-function mutant IDH1 (mut. IDH1) that reduces α-KG to the oncometabolite 2-hydroxyglutarate (2-HG), consuming NADPH in the process. The inhibitors ivosidenib and olutasidenib potently bind mut. IDH1. Ivosidenib also inhibits w.t. IDH1 at an IC50 of 24–71 nM, whereas the IC50 of olutasidenib for w.t. IDH1 is 22,400 nM, indicating no inhibitory activity

Inhibition of wild-type IDH1 has implications for its downstream metabolic functions. The normal function of wild-type IDH1 is illustrated in Fig. 2. Wild-type IDH1 enzyme catalyzes the oxidative decarboxylation of D-isocitrate to α-ketoglutarate (α-KG). Inhibition of wild-type IDH1 in cells can deplete α-KG and by extension NADPH. Since the reductive power of NADPH is protective against reactive oxygen species (ROS), reduced NADPH production leads to increased oxidative stress [17]. It has been shown that IDH1 knockout (KO) mice have increased levels of ROS in the liver compared to wild-type mice, which affected their survival after sublethal lipopolysaccharide injection [18]. Furthermore, changes in NADP + /NADPH ratio and associated ROS have been linked to ion channel dysfunction, which may have implications on QT prolongation. It is interesting to note that QT prolongation was observed in vivo for ivosidenib. The QTc predicted prolongation observed in patients treated with ivosidenib was 16.1 ms [19]. In contrast, the largest mean increase in QTc interval observed for olutasidenib was 6.2 ms [20]. In clinical trials, the incidence of grade 3 or higher prolongation of QT interval as measured by ECG was 7.8% (14/179) in the R/R AML overall population who received ivosidenib, and < 1% (1/153) in the R/R patients who received olutasidenib.

Depletion of α-KG also has oncogenic consequences as it is a rate-limiting substrate of 2-oxoglutarate-dependent dioxygenases and is an essential cofactor for histones and DNA demethylases. Additionally, α-KG is required for amino acid driven gluconeogenesis, the loss of which is linked to poor cell viability in low glucose conditions and worsened survival in animals under fasted conditions [21]. Alpha-ketoglutarate also plays a role in erythropoiesis by affecting heme production. It is apparent that wild-type IDH1 maintains several important functions that can affect cell viability and survival under stress conditions. Therefore, treatments with greater selectivity towards the mutant form of IDH1 without inhibiting the normal function of wild-type IDH1 may have clinical benefits without undue effects on hematopoiesis.

### Molecular Binding of Olutasidenib and Ivosidenib to IDH1

As with most mIDH1 inhibitors, olutasidenib and ivosidenib do not bind at the active site of each protein monomer, but instead bind to the interface of an IDH1 dimer. The dimer interface forms an allosteric site demarcated in part by alpha helices (alpha helix 10 from each monomer) which are adjacent to the catalytic site in both mutant and wild-type IDH1 monomers. The normal IDH1 enzymatic activity of converting isocitrate to α-KG with corresponding reduction of NADP + to NADPH requires wild-type IDH1 homodimers [22]. In cell culture and in vitro assays, both mutant IDH1 homodimers and wild-type/R132 IDH1 heterodimers demonstrate the ability to catalyze reduction of α-KG to 2-HG with consumption of NADPH [23, 24]. However, in cancer cells bearing a mutant *IDH1* R132 allele, the wild-type/R132 heterodimer configuration is considered the predominant driver of neomorphic activity, possibly due to its more abundant form, availability of α-KG substrate, or in vivo efficiency [25, 26].

Olutasidenib has a lower molecular weight than ivosidenib (FW 355 vs FW 583, respectively) and occupies a smaller space within the allosteric pocket formed by an IDH1 dimer. The smaller size of olutasidenib allows it to bind independently to each monomer unit of IDH1 within the dimer interface, potentially at a 2:1 ratio of olutasidenib to IDH1 dimer. In contrast, due to its larger size, a single ivosidenib molecule straddles the allosteric pocket of the dimer, resulting in a ratio of 1 molecule of ivosidenib per IDH1 dimer, regardless of whether the dimer is composed of wild-type homodimer, wild-type/R132 heterodimer, or R132 IDH1 homodimer [27].

The binding properties of the 2 molecules have implications for how each drug affects interactions and kinetics within the binding pocket of either mutant or wild-type IDH1 dimers. Both molecules form interactions with Asp279, found on the alpha 10 helix that makes up one side of the allosteric binding pocket. This same alpha helix sits in between the allosteric binding site and the catalytic site. Notably, residues from alpha 10 helix normally facing the catalytic site form interactions with a magnesium ion via Asp279, Asp275, and Asp252. The magnesium ion binding plays a key role in enzyme catalytic reaction. Both olutasidenib and ivosidenib interact with Asp279, which pulls away the aspartate residue from interacting with the magnesium ion. The weakened magnesium ion binding is thought to be the mechanism by which both inhibitors target mutant and wild-type IDH1 enzymatic activity.

Reports indicate that ivosidenib binds slowly and tightly to wild-type IDH1. It is possible that ivosidenib stays bound longer and occupies the allosteric pocket in a more constant state, which may result in an increased interaction with wild-type IDH1 and its subsequent inhibition. This is consistent with ivosidenib binding to both wild-type and mutant IDH1 in isothermal calorimetry (ITC) assays [28]. In contrast, the smaller olutasidenib molecule has weaker binding affinity to wild-type IDH1, making it a more selective inhibitor of mutant IDH1.

### Differential Activity Against IDH1 Double Mutants

**Fig. 3:**
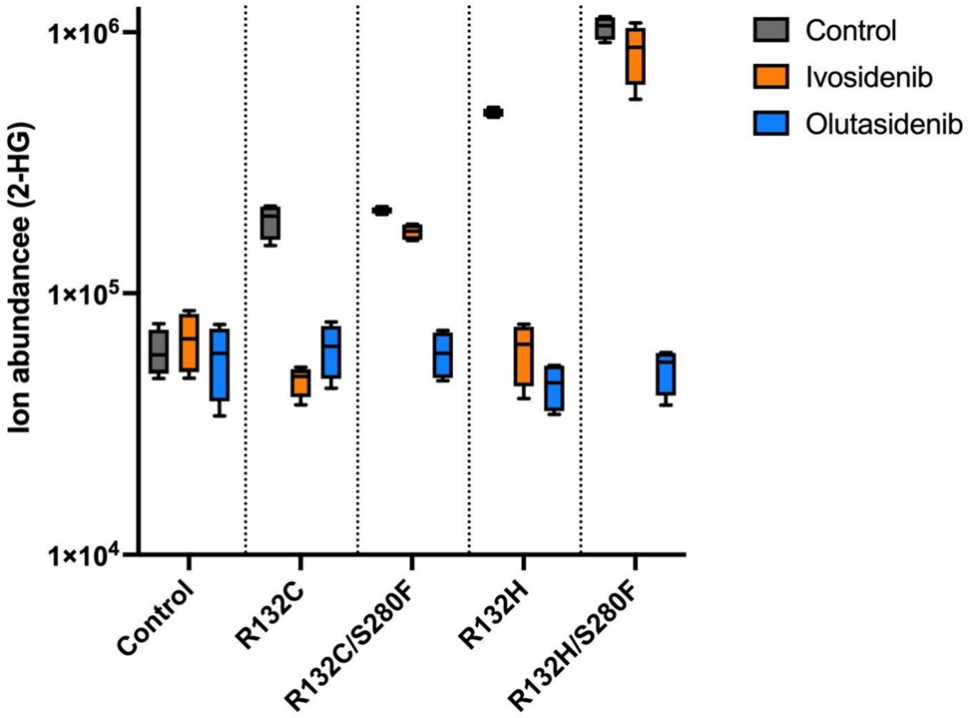
Influence of inhibitors on 2-HG levels in cells bearing IDH1 mutations [27]. (Adapted from Reinbold et al. [27].) LN18 cells were treated with the inhibitors ivosidenib, olutasidenib in DMSO (final concentrations: 5 μM). 2-HG levels were determined by anion-exchange chromatography couple to MS (*n* = 4 independent replicates). Control cells were generated by transduction with lentiviral vectors containing no IDH1. Box-and-whisker plots: The center line is the median and the bounds are 25th and 75th percentile values. The whiskers are the min and max measured 2-HG levels for each experimental group

**Fig. 4:**
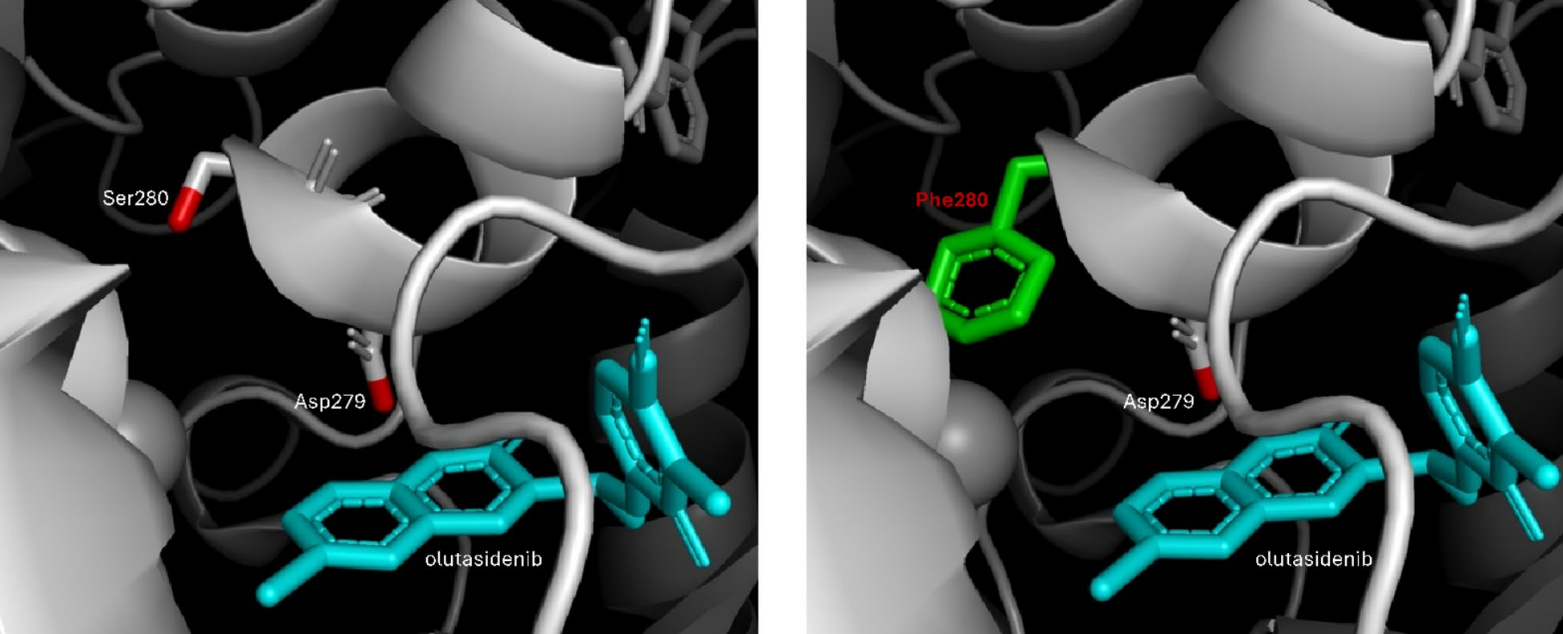
Binding of olutasidenib in single- or double-mutant IDH1. Left: Crystal structure of the binding pocket of single-mutant IDH1 R132H. Olutasidenib (blue) interacts with Asp279 in the IDH1 dimer interface. There is ample space remaining near the adjacent Ser280 site. Right: Generated image of the binding pocket of double-mutant IDH1 R132H/S280F based on the crystal structure of IDH1 R132H and grafting on the phenylalanine substitution. In the presence of a second site mutation whereby the serine is replaced with phenylalanine (S280F), the bulky benzene ring of phenylalanine increases steric hindrance and prevents larger molecules from interacting with Asp279 in the binding pocket. Due to its small size, olutasidenib is able to bind Asp279 even in the presence of the second site S280F mutation

Choe et al. first reported that the acquisition of a second-site mutation is one potential mechanism of ivosidenib resistance [29]. Reinbold et. al. further screened IDH1 inhibitors, including ivosidenib and olutasidenib, for inhibitory activity against the *IDH1 R132* hotspot mutation, with or without a second site mutation *in cis* [27]. It was reported that although ivosidenib demonstrated potency against IDH1 single-mutation variants R132C and R132H, no inhibition was detected against the R132C/S280F and R132H/S280F double-mutation variants [27]. In contrast, olutasidenib retained binding affinity to both single- and double-mutated IDH1 variants (Table 3) [27]. Furthermore, in a cellular assay, ivosidenib failed to suppress 2-HG production in R132C/S280F or R132H/S280F-positive cells whereas olutasidenib reduced 2-HG levels in the same cell types (Fig. 3; adapted from Reinbold et al.) [27]. Structural analysis indicated that the increased size of the phenylalanine in S280F, adjacent to the key Asp279 residue, blocks binding of ivosidenib via steric hindrance. Interestingly, the crystal structure of the smaller olutasidenib bound to IDH1 suggests that it can accommodate the bulky phenylalanine, consistent with biochemical data (Fig. 4).

**Table 3:** IC_50_ values^a^ (UV-based assay), nM [27]

	R132C	R132C/S280F	R132H	R132H/S280F
Ivosidenib	2.5 ± 0.1	No inhibition observed	2.9 ± 0.3	No inhibition observed
Olutasidenib	120 ± 8	(1.33 ± 0.03)*10^3^	10 ± 0.7	76 ± 2

^a^IC_50_ values (standard error of the mean, *n* = 3) with IDH1 inhibitors (at 30 nM)

Adapted from Reinbold et al. [27]

## Clinical Implications of Molecular Differences

We have established that although olutasidenib and ivosidenib both target mutant IDH1, it is clear they are not the same molecule nor have the same selectivity. It is of clinical interest to know if patients may respond differently to the two FDA approved mIDH1 inhibitors, which have distinct chemical structures and binding properties. There are observed differences in clinical outcomes upon examining the respective clinical trials with these two therapies in comparable AML populations, but to date, comparisons remain limited in the absence of a head-to-head study.

In the olutasidenib pivotal trials evaluating safety and efficacy in patients with m*IDH1* AML, multiple cohorts were enrolled based on their prior treatment and olutasidenib was evaluated as both a single agent (pivotal cohort and sub-cohorts) and in combination with azacitidine (sub-cohorts). This afforded the opportunity to analyze a subgroup of AML patients who were relapsed or refractory (R/R) to prior ivosidenib treatment and then received olutasidenib in the trial. The subgroup analysis of R/R ivosidenib patients included patients from 2 cohorts in the phase 1 dose-escalation/dose-expansion portion of the study and 6 out of 8 cohorts from the phase 2 portion of the study. Patients received either monotherapy with olutasidenib (150 mg QD or 300 mg QD or 150 mg BID) or combination therapy with 150 mg olutasidenib + 75 mg/m^2^ azacitidine. Response to treatment was defined by the modified IWG criteria 2003, with rate of CR + CRh as the primary efficacy endpoint.

Of 355 R/R patients in the olutasidenib study, 9 patients had prior ivosidenib. Of the 9 patients who had failed ivosidenib before entering the trial, 6 had relapsed within 12 months of their prior treatment before entering the olutasidenib study. In the olutasidenib study, 4 patients received olutasidenib monotherapy and 5 received the combination regimen of olutasidenib and azacitidine. Notably, 2 of the patients who failed prior ivosidenib were able to achieve complete remission on olutasidenib combination treatment, one of whom went on to receive HSCT. One patient had 2 prior treatments before receiving olutasidenib, including an HMA (decitabine) followed by ivosidenib. This patient achieved a CR on olutasidenib for 5.6 months before progressing. The second patient had 3 prior treatments, including cytarabine, followed by a cytarabine, etoposide, mitoxantrone combination, followed by ivosidenib. This patient achieved a CR on olutasidenib and proceeded to HSCT after 3.1 months. Adverse events in this subgroup of patients were consistent with what was previously reported for patients undergoing treatment for AML.

## Discussion

Since the approval of olutasidenib in December 2022, there are now two viable targeted therapies for patients with mutant *IDH1*-positive AML*.* Both therapies inhibit mIDH1, provide sustained 2-HG suppression, and demonstrate similar CR/CRh rates [7, 8]. However, differences were observed in clinical outcomes on the pivotal trials for each agent, namely, a longer duration of response (CR and CR/CRh), improved survival in responders, and a higher rate of complete remission among patients who received olutasidenib therapy [7, 8]. In addition, we examined patients with R/R AML in the multi-cohort phase 2 olutasidenib trial who had received prior ivosidenib treatment. Interestingly, there were 2 patients who were able to achieve complete remission on olutasidenib who were either relapsed or refractory to prior ivosidenib, including one who was relapsed from a prior HMA. Therefore, it appears that there are different clinical outcomes among patients who receive the two treatments despite the same target. We explored the potential explanations for the observed clinical differences in this review.

From a molecular perspective, olutasidenib and ivosidenib were identified through unique molecular screens and thus are structurally very different molecules [27]. Molecular structure translates to selectivity differences. Namely, olutasidenib selectively inhibits mutant but not wild-type IDH1, whereas ivosidenib appears to potently block both mutant and wild-type IDH1 [15, 29]. Inhibiting wild-type IDH1 may cause off-target effects, as wild-type IDH1 is essential for cell viability and homeostatic functions. Moreover, blocking wild-type IDH1 may induce oxidative stress, which may weaken the immune response and provoke clonal evolution, which could lead to therapy resistance [18, 21]. Olutasidenib inhibitory activity may have a “goldilocks” effect by binding tightly enough to mutant IDH1 to suppress 2-HG but not so tightly as to also inhibit wild-type IDH1. By virtue of not inhibiting wild-type IDH1, olutasidenib is a more selective mIDH1 inhibitor compared to ivosidenib.

Limitations of this review include the lack of a randomized head-to-head trial of ivosidenib versus olutasidenib and the small subgroup analysis conducted on the patients who had received both ivosidenib and olutasidenib. As such, the review can only compare and discuss results from two independent trials. A formal comparison would require a randomized trial so that factors such as study design and patient population can be properly controlled. However, the structure and binding mechanisms discussed herein provide a potential rationale for the notable difference in clinical outcomes observed in patients with R/R mIDH1 AML who were treated with olutasidenib or ivosidenib. Clinical trials exploring switch maintenance and triplet therapy with venetoclax + hypomethylating agent + olutasidenib or ivosidenib are planned or underway in multiple patient populations with mIDH1 AML. With expanding use of both olutasidenib and ivosidenib in the real-world and in a variety of settings, more distinguishing factors may arise to further define how to best treat patients with myeloid and other malignancies harboring an *IDH1* mutation.

## Key References

- de Botton S, Fenaux P, Yee KWL, et al. Olutasidenib (FT-2102) induces durable complete remissions in patients with relapsed or refractory IDH1-mutated AML. Blood Adv 2023.
  - ○ This reference is of outstanding importance because it describes the pivotal cohort that led to the FDA approval of olutasidenib for the treatment of relapsed or refractory AML with an IDH1 mutation.
- DiNardo CD, Stein EM, de Botton S, et al. Durable Remissions with Ivosidenib in IDH1-Mutated Relapsed or Refractory AML. N Engl J Med 2018; 378:2386–98.
  - ○ This reference is of outstanding importance because it describes the efficacy and safety of ivosidenib in patients with relapsed or refractory, IDH1-mutant AML.
- Cortes J, Jonas BA, Schiller G, et al. Olutasidenib in post-venetoclax patients with mutant isocitrate dehydrogenase 1 (mIDH1) acute myeloid leukemia (AML). Leuk Lymphoma 2024:1–8.
  - ○ This reference is of importance as it presents the effectiveness of olutasidenib in patients who were relapsed or refractory to venetoclax and thus have a notably poor prognosis.
- Reinbold R, Hvinden IC, Rabe P, et al. Resistance to the isocitrate dehydrogenase 1 mutant inhibitor ivosidenib can be overcome by alternative dimer-interface binding inhibitors. Nat Commun 2022; 13:4785.
  - ○ This reference is of outstanding importance because it describes the binding properties and inhibitor activity of IDH1 inhibitors such as olutasidenib and ivosidenib against single mutant and double mutant IDH1 acute myeloid leukemia cells. The occurrence of a second mutation which produces IDH1 R132C/S280F causes ivosidenib resistance. Olutasidenib retains inhibitor activity in the presence of the second-site mutation.

## Data Availability

No datasets were generated or analysed during the current study.
